# Low prevalence of HIV in the northern Cameroon: contribution of some AIDS restriction genes and potential implications for gene therapy

**DOI:** 10.3389/fgene.2024.1447971

**Published:** 2024-09-13

**Authors:** Patrice Djataou, Marceline Djuidje Ngounoue, Carine Nguefeu Nkenfou-Tchinda, Marie Nicole Ngoufack, Elise Elong, Aline Tiga, Clifford Muluh, Joelle Kadji Kameni, Moussa Djaouda, Alexis Ndjolo, Celine Nguefeu Nkenfou

**Affiliations:** ^1^ Chantal BIYA International Reference Center (CBIRC), Yaounde, Cameroon; ^2^ Department of Biochemistry, Faculty of Science, University of Yaounde I, Yaounde, Cameroon; ^3^ Department of Biochemistry, Faculty of Medicine and Pharmaceutical Sciences of Sangmelima, Ebolowa University, Sangmelima, Cameroon; ^4^ UNICEF (Cameroon), Yaounde, Cameroon; ^5^ Department of Life and Earth Sciences, University of Maroua, Maroua, Cameroon; ^6^ Department of Biological Sciences, Higher Teachers Training College, Yaounde, Cameroon

**Keywords:** AIDS restriction host genes, gene therapy, HIV, Northern Cameroon, risk factors, surveillance

## Abstract

**Background:**

HIV infection and its progression to AIDS depend on several factors including host genetic factors. The immunological mechanisms of host resistance to HIV infection greatly influence the prevalence of HIV in a given region. Worldwide, Cameroon not exempted, the frequency of AIDS-associated genes varies and may influence this prevalence. The North and Far North Regions of Cameroon have had the lowest HIV prevalence in the country for many years despite risky behaviors associated with their customs and habits. In this work, we seek to explore the contribution of host genes to the HIV low prevalence in these regions.

**Methodology:**

Five genes variants previously described as HIV AIDS related were studied. These genes are: *CCR5Δ32*, *CCR5promoter59029G*, *CCR2-64I, SDF1-3ʹA* and *Trim5α(R136Q)*. A total of 384 consented participants were included in this study. The HIV serological status was confirmed using national algorithm. Genomic DNA was extracted from the buffy coats and used for genotyping. The results obtained were compiled in Excel 2016, Epi Info 7.1 and snpStats software and Chi two tests allowed us to compare the frequencies of the AIDS related alleles in the North with those in other Regions of Cameroon and to measure the impact of these ARGs on protection against HIV.

**Results:**

The frequency of protective alleles *CCR5Δ32, CCR5promoter59029G, CCR2-64I, SDF1-3ʹA* and *Trim5α(R136Q)* was the allelic frequencies should be expressed as percentages i.e. 0.52%; 37.56%; 36.46%; 25.19% and 69.33%. These allelic frequencies exhibited a significant difference when compared to those obtained in other regions of Cameroon (*p < 0.01*). Protective alleles were predominant in the Northern region compared to others and were associated with resistance to HIV [(*p < 0.0001*); OR = 2.02 CI, 95%].

**Conclusion:**

The higher frequency of HIV-protective alleles in the northern regions may be a contributing factor to the lower prevalence of HIV. Nevertheless, this should be reinforced by other preventive and surveillance methods to guarantee the sustained low prevalence. HIV can develop resistance through the process of mutation, but the host targets themselves are genetically stable. The study of these host genetic restriction factors is of great value in the design of a practical cure for HIV infection or an effective vaccine.

## Introduction

Many years after its discovery, HIV/AIDS remains a major public health problem worldwide. The pandemic has spread rapidly around the world, with a peak in the number of infections recorded in the mid-1990s (Lot, 2008). Today, HIV pandemic is more or less under control, with an overall decline in prevalence and new infections worldwide (UNAIDS, 2024). Preventive measures, awareness raising and antiretroviral treatments are helping to significantly reduce the number of new infections each year. Current treatments aim to limit viral replication and reduce the viral load to an undetectable level (Ahmed et al., 2022; Abrahams et al., 2009); while controlling the emergence of ARV drugs resistance. As a result, the life expectancy of people living with HIV has improved significantly. Research into HIV/AIDS has evolved, and today science already has the means to control the infection with new-generation ARVs. While HIV/AIDS awareness and prevention in communities have produced satisfactory results, research into a definitive vaccine remains a challenge for researchers. In the HIV infection process, chemokine co-receptors (CCR5 or CXCR4) interact with gp120 to fuse with the membrane and integrate the target cell. Several genes have been correlated with the evolution of HIV within populations. Antiretroviral treatment has brought many advances in the fight against HIV, as well as improving the life expectancy of seropositive people (Burgoyne and Tan, 2008). However, with the emergence of resistance to ARVs, the search for new curative technologies remains a challenge. Resistance to ARVs increases the risk of transmission of the virus with anticipated rapid progression of the disease to the AIDS stage because the virus has become resistant to the treatment (Sayan et al., 2016). In fact, studies show a mutation in the selection of viral tropism from R5 to the more virulent R4 (Yandrapally et al., 2021). HIV-associated genes remain a real mine of research because they influence the mechanisms of infection in the primary stages, pathogenesis and response to antiretroviral treatment.

In the history of HIV/AIDS, two cases of remission have been recorded: one in Berlin and the other in London (Gupta et al., 2019; Gupta et al., 2020a). These two patients have received through transplantation HIV-associated resistance gene, *CCR5Δ32*, which has been associated for many years as host gene for resistance to infection. In host cell infection mechanisms, HIV recruits in addition to the CD4 receptor, a CCR5 coreceptor to fuse with the host membrane. A particular mutation of the *CCR5* gene co-receptor to *CCR5Δ32* characterized by a deletion of 32 base pairs in its coding region prevent the virus from infecting the cell (Agrawal et al., 2004). After biosynthesis, this mutation produces a truncated receptor that is incapable of being recruited by HIV, so there is no infection. SDF-1 (stromal derived factor-1) is the only known ligand for CXCR4 (Sadri et al., 2022). Binding of SDF1 to CXCR4 leads to internalization of the receptor (Arenzana-Seisdedos, 2015). Thus, the virus with “R4″ tropism cannot bind the receptor and therefore penetrate the target cell. In the homozygous state, *SDF1-3′A/A* delays the onset of acquired immunodeficiency syndrome (AIDS), according to a genetic association analysis of 2,857 patients enrolled in five AIDS cohort studies (Winkler et al., 1998). The recessive protective effect of *SDF1-3′A* was increasingly pronounced in individuals infected with HIV-1 for longer periods, was twice as strong as the dominant genetic restriction of AIDS conferred by CCR5 and CCR2 chemokine receptor variants in these populations, and was complementary with these mutations in delaying the onset of AIDS (Winkler et al., 1998). The mechanism of resistance to HIV infection would probably be due to the blockage of R5 to more aggressive R4 HIV-1 receptor switching leading to the AIDS stage (Brambilla et al., 2000). Studies have shown that the *SDF1-3′A* polymorphism has been associated with high plasma levels of SDF1 (Brambilla et al., 2000; Signoret et al., 1997). SDF1 induces internalisation of CXCR4 receptors, thereby preventing the selection mutation from R5 to R4 tropism (Yandrapally et al., 2021; Soriano et al., 2002). CCR2 is one of the most extensively studied genetic factors confirmed to be linked to host resistance to HIV-1 infection. A *CCR2-64I* point mutation (rs1799864) characterized by guanine substitution leads to the replacement of the amino acid valine (V) by isoleucine (I) at position 64 in the first transmembrane domain of CCR2. CCR2 can also, under certain conditions, be used as a co-receptor by HIV (Soriano et al., 2002). Numerous studies have reported that the *CCR2-64I* polymorphism delays the progression of HIV-1 to AIDS by maintaining a stable HIV viral load and a slow decline in CD4 cells (Ammaranond et al., 2011; Juhász et al., 2012). The mechanism of *CCR2-64I* protection can be explained by a direct modification of the kinetics of infection, or by an indirect effect of the physiological concentration of CCR2 on the availability of CCR5 on the surface of target cells, or by a regulatory binding imbalance in the regulatory or promotion region of this gene (Metodiev, 2012). In addition, studies have shown that the *CCR2-64I* mutant allele forms heterodimers with CXCR4, and may therefore impair the function of CXCR4 as a co-receptor for X4 viruses (Burton et al., 2005). Thus, individuals in various populations harboring *CCR2-64I* (Angela Covino et al., 2016), *CCR5Δ32* (Gupta and Padh, 2012), and *CCR5 promoter A/G* (Jang et al., 2008) mutations are less susceptible to HIV-1 infection and progress much more slowly to AIDS. The *CCR5* polymorphism results in the absence of cell surface CCR5 expression, whereas the *CCR2-64I* gene variant confers resistance to AIDS progression, probably due to the ability of this mutant receptor to heterodimerize with the CCR5 and CXCR4 receptors (Mellado et al., 1999).

*Trim5α* play a critical role in the primate anti-viral defence system. Trim5α in the Rhesus monkey completely blocks HIV-1 infection whereas human Trim5α mediates a low level inhibition to HIV-1 replication (Kim et al., 2019; Chan et al., 2014). The mechanism of HIV-1 restriction is not fully understood. Proposed models include binding of a multimer of Trim5α to the incoming viral capsid that leads to premature uncoating of the capsid or by interaction between Trim5α and the capsid with other undefined cofactors (Sokolskaja and Luban, 2006). Using an *in vitro* assay system, cells were transduced with different *Trim5α* missense polymorphisms; *R136Q* enhanced HIV restriction while *H43Y*, *V112F*, and *G110E* relaxed restriction on HIV replication or had no effect (*G249D, H419Y*) (Nakajima et al., 2009; Javanbakht et al., 2006; Goldschmidt et al., 2006; Sawyer et al., 2006).

This implies that there is a combination of genes that play a role in the resistance to HIV infection and that these genes must be addressed collectively.

HIV prevalence in Cameroon was estimated at 3.7% in 2018 (DHS cameroon, 2018). However, infection is unevenly distributed across the country. The North and Far North Regions of Cameroon have the lowest seroprevalence rates in Cameroon, at 1.6% and 1.5% respectively (DHS cameroon, 2018) for a national incidence rate of 0.24%. The low prevalence of HIV infection in this region is thought to be due to genetic factors that protect the population against infection. Cameroon is known for its ethnic and genetic diversity (Spínola et al., 2011). AIDS-Related Genes (ARG) genotyping studies have been conducted in the Central and Western Regions of Cameroon, revealing important information about the disease progression. (Nkenfou et al., 2013; Dambaya et al., 2019; Ma et al., 2005). However, these ARG genotyping studies have not yet been carried out in these two northern regions of Cameroon. In order to understand the uneven distribution of HIV seroprevalence in Cameroon, we will compare the distribution of ARGs and susceptibility to HIV infection in the Northern Regions and the Central and Western Regions of Cameroon.

## Material and methods

### Study framework and context

The study was carried out in the Garoua and Maroua Regional Hospitals located respectively in the North and Far North Regions of Cameroon. Samples will be collected between 15 June 2022 and 30 July 2022, following ethical approval and consent from participants.

### Sample size

The epi Info 7 calculation “STATCALC” software was used to calculate the population size (https://www.epivf.fr/calcul_nsn.html accessed July 2024) (Lwanga et al., 1991). We assumed that, given the low prevalence of HIV, the population had a high frequency of allelic ARGs of 50%, with an acceptable margin of error of 5%, 80% of power and a confidence level of 95%.

The population size is given by the following formula:

$$n=z2\text{xp}\left(1‐p\right)/e2\;/\;1+\left[z2\;x\;p\;\left(1‐p\right)/e2\;N\right]$$

Where n is the sample size.

N is the estimated size of the total population.
z is the 95% confidence level, p is 50%, the estimated proportion of ARGs in this population.
e the tolerated margin of error 5%.
That’s 384 participants enrolled.

### Sampling

#### Inclusion criteria

-People aged between 18 and 70 of both sexes who present at the hospital during the study;
-People who have been living in the North and Far North Regions and are native of these regions,
-Volunteers who have signed the informed consent form.

#### Eclusion criteria

Individuals whose data were incomplete, whose samples could not be analyzed due to insufficient quantity or quality, as well as those whose HIV status was indeterminate, were excluded from the study.

384 participants were enrolled after listening to/reading and signing the consent form to take part in the study and completing a questionnaire. Subsequently, 5 mL of blood in EDTA tubes was collected from each subject, and the buffy coat was separated after centrifuging the blood samples at maximum speed for 1 minute and pipetting the thin whitish layer on top of the red blood cells for the purpose of performing ARG genotyping. HIV serology tests were carried out using the national algorithm (parallel two tests). Reactive Determine HIV ½ tests were repeated using “KHB” test.

### DNA extraction and polymerase Chain reaction

The Buffy coats were used as a source of genomic DNA, that was extracted using QiaAmp DNA mini kit (Qiagen S.A. Three Avenue du Canada, LP 809, 91,974 Courtaboeuf Cedex, France), according to the manufacturer’s instructions. DNA concentration was measured by a nanodrop spectrophotometer (Thermo fisher, 3,411 Silverside Road Tatnall Building, Suite 100 Wilmington, DE 19810 U.S.A.).

The *CCR5Δ32*, *CCR5 promoter*, *CCR2-64I*, *SDF1-3ʹA* and *Trim 5α* genetic variants in participants were determined by PCR followed by RFLP detection using the specific primers and restriction endonucleases as described previously. Nevertheless, this original protocol was optimized during our study (Nkenfou et al., 2013; Ma et al., 2005; Kristiansen et al., 2001; Manen et al., 2008).

The amplification of *CCR5* was done as follows: one cycle for 30 s at 94°C, followed by 40 cycles of 30 s, 30 s, and 1 min at 94°C, 50°C, and 72°C, respectively, followed by a final extension of 10 min at 72°C. *CCR5-promoter* gene amplification was done as follows: 3 min at 94°C, followed by 40 cycles of 30 s, 30 s, and 45 s at 94°C, 60°C, and 72°C, respectively, and a final extension of 10 min at 72°C. *CCR2* gene was amplified in one cycle of 30 s at 94°C, followed by 40 cycles of 30 s, 30 s, and 30 s at 95°C, 63°C, and 72°C, respectively, and a final extension of 10 min at 72°C. To detect *SDF-1* gene, the amplification started with a denaturation step of one cycle of 3 min at 94°C, followed by 40 cycles of 30, 30, and 30 s at 94°C, 58°C, and 72°C, respectively, and a final extension of 10 min at 72°C. The amplification of *Trim 5α* gene fragment was done using the following conditions: one cycle of 3 min at 94°C, followed by 40 cycles of 30 s, 30 s and 1 min at 94°C, 50°C and 72°C, respectively, and a final extension of 10 min at 72°C (Sawyer et al., 2006; DHS cameroon, 2018).

The amplified fragments were run in agarose gel with variable percentage (1%–1.5%) depending on the fragment size, previously stained with ethidium bromide, and visualized under ultraviolet light. The above-mentioned gene fragments size and their specific primers are presented in (Table 1).

**TABLE 1:** Primers used and expected fragments size of studied genes.

Gene variants	Primers’ sequences	AS (pb) (ESED (bp))	Enzymes	References
*CCR5∆32*	5′-CTTCATCATCCTCCTGACAATCG-3′ 5′-GACCAGCCCCAAGTTGACTATC-3′	262 or 230 (NA)	None	Kristiansen et al. (2001), Köksal et al. (2021)
*CCR2-64I*	5′-GGATTGAACAAGGACGCATTTCCCC-3′ 5′-TTGCACATTGCATTCCCAAAGACCC-3′	380 (380, 215, 165)	*Fok* I	Nkenfou et al. (2013), Köksal et al. (2021)
*SDF1-3′A*	5′-CAGTCAACCTGGGCAAAGCC-3′ 5′-AGCTTTGGTCCTGAGAGTCC-3′	302 (302, 202, 100)	*Msp* I	Nkenfou et al. (2013), Kristiansen et al. (2001)
*Trim5α(138Q)*	5′-ATGGCTTCTGGAATCCTGGTTAATG-3′ 5′-CCCGGGTCTCAGGTCTATCATG-3′	526 (526, 405, 121)	*Ava* I	Dambaya et al. (2019)
*CCR5proA/G*	5′-TGGGGTGGGATAGGGGATACTGTATT-3′ 5′-GAAGGCGAAAAGAATCAG-3′	498 (498, 435, 45)	*Bsp 1286* I	Nkenfou et al. (2013), Nkenfou et al. (2021)

AS, is amplicon size; ESED, expected size after enzyme digestion.

#### Genotypic analyses

Genotyping was carried out with the use of restriction fragment length polymorphism (RFLP) method except for the *CCR5-Δ32* deletion based on the respective restriction enzyme sites in the four amplified (PCRs) products of *CCR5 promoter, Trim 5α, CCR2* and *SDF1* as described previously, (Table 1). The restriction enzymes used in this work were purchased from Thermo Fischer and used according to manufacturer instructions.

#### Statistical analyses

Allele and genotype frequencies and the Hardy-Weinberg equilibrium (HWE) were evaluated using SNPstats software (snpStats). All alleles achieved HWE. The differences in the allele frequency of each genetic variant between and within the distinct groups of HIV-1 seronegative and HIV-1 seropositive individuals in Northern and Central, West, and other Cameroon regions were determined by Chi-square or Fisher exact test when indicated. To estimate the association between ARG polymorphisms and participants’ serology, we used several inheritance models (codominant, dominant, recessive, overdominant and additive). For each ARG, odds ratios (OR) and 95% confidence intervals (CI) were calculated using logistic regression analysis.

*p*-values were calculated, and *p < 0.05* was considered statistically significant.

#### Ethical considerations

The National Ethics Committee reviewed the protocol for ethical consideration and approval was given under N°2021/06/85/CE/CNERSH/SP. Informed consent or assent/parental consent form was signed when applicable by all the participants before enrolment. As well, this study was conducted in accordance with the Declaration of Helsinki (WMA).

## Results

A total of 384 participants were enrolled, regardless of gender, with a mean age of 31.25 years and a standard deviation of 10.2 years. Table 2 presents the sociodemographic characteristics of the study population. The most common age group was between 18 and 35 years. Females comprised n = 203 (52.86%) of the subjects in our study population compared to n = 181 (47.14%) males, a ratio of 1.1 in favor of females. The overall HIV prevalence in this study population was 1.63%. Some participants admitted to having had a sexually transmitted infection such as syphilis (n = 21), gonorrhea (n = 37) and *chlamydia* (n = 12).

**TABLE 2:** Socio-demographic characteristics of the study population.

	Men	Women	*p-value*
Participants	181 (47.14)	203 (52,86%)	*0.8*
Average age	31.74	30.84	*0.77*
Religion • Muslim • Christian	65 116	66 137	*> 0.42*
Level of education • University • Secondary • Primary/Coranic • Illiterate	91 58 29 3	93 70 30 10	*> 0.5*
Marital status • Married • Single • Divorced/widowed • Cohabitation	79 01 04 97	98 15 09 81	*0.7*

### Distribution of the five genes variants in the study population

In the overall population, *CCR5∆32* double mutation (homozygote mutants) was completely absent in the study population and 1.04% for heterozygous genotype. The double mutation G/G of *CCR5 promoter* was frequent at 66.93%, with 20.83% for heterozygous genotype.

*SDF1-3ʹA* double mutation was 2.08%, and the heterozygous genotype was present at 97.92%.

Homozygous wild type for *SDF1-3ʹA* were absent. The double mutation of *Trim5α* Q136 was 61.72% (Table 3).

**TABLE 3:** Distribution of genotypes and frequencies of ARGs protective allele (*CCR5Δ32, CCR2-64I, SDF1-3ʹA, CCR5 Promoter 59029G* and *Trim 5α(138Q)*) in the study population.

Gene variants	Genotypes	N	Genotypes frequencies (%)	Frequency of protective allele (%)
***CCR5*** *Δ32*	Wt/wt Wt/Δ32 Δ32/Δ32	380 04 00	98.96 1.04 0.0	0.52
***CCR2-*** *64I*	64V/64 V 64V/64I 64I/64I	49 260 75	12.76 67.71 19.53	36.46
***SDF1-*** *3′A*	3′G/3′G 3′G/3′A 3′A/3′A	42 327 15	10.94 85.16 3.9	25.19
***CCR5promoter59029G***	A/A A/G G/G	47 257 80	12.24 66.93 20.83	37.56
***Trim5α (*** *136Q)*	R136/R136 R136/Q136 Q136/Q136	12 141 231	3.12 36.72 60.16	69.33

Abbreviations: wt, wild type: The allelic frequencies calculated here are for the protective allele (*CCR5-Δ32, CCR5 promoter 59029G, CCR2-64I, SDF1-3ʹA and Trim5α (136Q)*.

The heterozygous genotype for the *CCR5-Δ32* mutation was present in our study population although rare or absent in Africa according to several data. This genotype was found in the Moundang (n = 3) and in a Massa (n = 1) tribes in our study population. These two ethnic groups share the same department of origin. It is therefore possible that the homozygous *CCR5-Δ32* mutation is present in this locality.

### Distribution of genetic variants according to serological HIV status in the study population

The most represented alleles for *CCR5* variant was the wild type (wt), for *CCR5p59029* variant it was *CCR5p-59029A*; for *Trim5α*, it was *Trim5α (136Q)*; for *CCR2* variant the major allele was *CCR2-64I* and for *SDF1* it was *SDF1-3′A*.

*Trim5α(136Q)* (OR = 3.3 [CI, 95%; 2.74–3.85]), *SDF1-3ʹA* (OR = 2.2 [CI, 95%; 1.52–2.87]) and *CCR5p-59029A* (OR = 2.6 [CI, 95%; 1.98–3.22]) may be strongly associated with resistance to HIV infection. Meanwhile, *CCR2-64I* (OR = 1.7 [CI 95%; 0.93–2.47]) and *CCR5∆32* (OR = 1.3 [ 95% CI; 0.42–2.18]) were less strongly associated with HIV infection (Table 4).

**TABLE 4:** Distribution of the genotypes of the 5 AIDS related genes variants in the study population according to HIV status.

Gene variants	Genotypes	HIV status		Odds ratios CI, 95%
		HIV-	HIV+	
***CCR5*** Δ32	Wt/wt Wt/Δ32 Δ32/Δ32	373 04 00	07 00 00	1.3 [0.42–2.18]
***CCR2-*** 64I	64V/64 V 64V/64I 64I/64I	47 256 74	02 04 01	1.7 [0.93–2.47]
***SDF1-*** 3′A	3′G/3′G 3′G/3′A 3′A/3′A	08 369 00	05 02 00	2.2 [1.52–2.87]
***CCR5promoter 59029G***	A/A A/G G/G	41 256 80	04 03 00	2.6 [1.98–3.22]
***Trim5α*** (136Q)	R136/R136 R136/Q136 Q136/Q136	07 139 231	05 02 00	3.3 [2.74–3.85]

wt, wild type.

The odds ratio for HIV, infection is measured by taking into account the allelic frequency of the wild-type gene compared with mutant variants of the same gene. The greater the odds ratio (greater than 1), the stronger the protective effect of the gene, and *vice versa*.

The search for a simple association between the presence of an ARG and the onset of HIV using SNPstats online software enabled us to show the protective effect of *Trim 5α(136Q)* (*p < 0.0001*) *CCR5 Promoter 59029 G* (*p < 0.001*), *CCR2-64I* (*p = 0.022*) and *SDF1-3ʹA* (*p < 0.001*). In addition, the combination of these ARGs showed a more pronounced protective effect against HIV acquisition (*p < 0.00001*).

### ARG frequency comparison between northern regions and other regions of Cameroon

**FIGURE 1:**
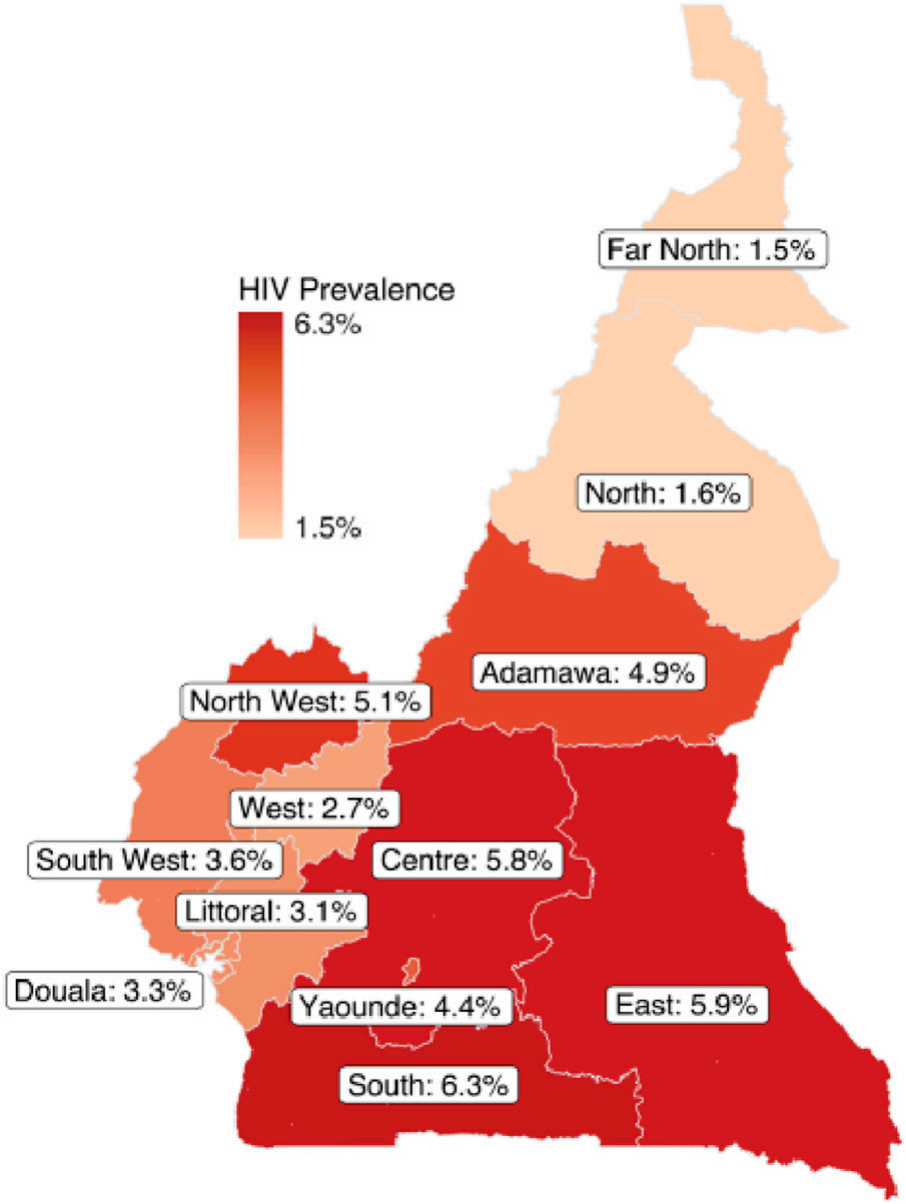
Cameroon map showing the distribution of HIV prevalence according to regions in 2020. https://phia.icap.columbia.edu/wp-content/uploads/2017/02/CAMPHIA-Summary-Sheet-EN_ARV-adjusted_Feb2020.pdf.

Multitude of studies on ARGs have been conducted in various regions of Cameroon. It was important to compare the allelic frequencies of the ARGs studied to those of other regions, as in Cameroon HIV prevalence va ries according to regions (see Figure 1). The study revealed a significantly higher prevalence of protective gene variants in the study region compared to other regions in Cameroon. Table 5 presents the distribution of ARG frequencies in other parts of the country in comparison to the northern Regions of Cameroon.

**TABLE 5:** Comparison of ARG frequencies between the Northern Regions and other Regions of Cameroon.

ARG genes variants	Alleles	Allelic frequencies in some Regions of Cameroon (%)					*p-value*
		West N = 179 (Nkenfou et al., 2013)	Other Regions N = 570 (Dambaya et al., 2019)	Adamawa N = 217 Unpublished data	Center N = 96 Nkenfou et al. (2021)	Northern N = 384	
*CCR5* Δ32	Δ32	00	00	00	00	0.52	<0.01^x,y,z,t^
*CCR2-*64I	64I	17.6	1.78	4.15	15.30	36.46	<0.01^x,y,z,t^
*SDF1-*3′A	3′A	27.93	5.35	15.66	24.71	25.19	<0.01 ^y,z^; = 0.22^xt^
*CCR5pro59029G*	G	49.7	20.84	NA	26.30	37.56	= 0.83^a^; = 0.03^y^; 0.019^t^
*Trim5α* (136Q)	136 Q	NA	42.26	NA	NA	69.33	<0.0001 ^y^

^a^ ^,y,z,t^ represents the *p-value* comparing frequencies in West, others Regions within Northern, Adamawa and Center *versus* Northern Cameroon. statistically, genes associated with resistance to HIV, infection such as *Trim5α (Q136/Q136)*; *SDF1-(3′A/3′A)*; *CCR2(64I/64I); CCR5(Wt/Δ32)* were significantly more frequent in northern Cameroon. The Adamawa region shares the most cultural and geographical similarities with the other two regions of northern Cameroon. however, the prevalence rates in the regions of northern Cameroon compared to Adamawa are statistically higher *p < 0.001*. The frequency of ARGs, could explain this seroprevalence.

NA: Not available.

#### Hardy-weinberg equilibrium

The overall analysis of genotype data did not show any deviation from the Hardy-Weinberg expected frequency, the χ^2^ tests showed that all genes’ loci in HIV infected or uninfected are in equilibrium (*p = 0.48*). The observed genotype frequencies had no significant difference from the frequencies expected in each group, indicating that the five alleles are effectively in genetic equilibrium.

## Discussion

The overall prevalence of HIV infection in this study was 1.82%. This prevalence was not statistically different from that published by the DHS in 2018 (*p* = 0.88), namely, 1.5% and 1.6% for the North and Far North regions of Cameroon (DHS cameroon, 2018). The northern regions have a high proportion of Muslims, and polygamy is permitted within this religious tradition. In light of the resurgence of other sexually transmitted infections (STIs) in Cameroon (Nkenfou et al., 2018), which are known to be risk factors for HIV, there is a potential for an increase in the transmission of HIV.

This contributes to the elevated prevalence of hepatitis B in these regions, which can be attributed to customary practices of sharing food items, such as eating from the same dish. Furthermore, a comparable study conducted by Mboppi and colleagues in a district of southern Cameroon indicated that muslim religion was associated with a high prevalence of sexually transmitted infections (STIs) and HIV infection (Mbopi-Keou et al., 2014). It is likely that these factors, including polygamy, early marriage, low rates of condom use, and poverty, may contribute to the high prevalence of STIs and HIV in this region. In addition, our study was carried out in hospital settings and not in the community. In addition, these regions have been subjected to armed conflict for an extended period, resulting in an influx of refugees (BANQUE MONDIALE). This could potentially compromise the resilience of the health system and contribute to an increase in HIV cases (Mishra et al., 2021).

The genotypes for the mutant *CCR5Δ32* gene were 98.96%, 1.04% and 00% respectively for wild-type, heterozygous and homozygous mutants, with an allelic frequency of the protective gene of 0.52%. This distribution corroborates several studies conducted in Cameroon and Africa where the Δ32 mutation is very rare or even absent (Nkenfou et al., 2013; Dambaya et al., 2019; Ma et al., 2005; Nyambi et al., 2002). However, studies conducted in Cameroon have demonstrated that this mutation is completely absent in the Central, Western, and Eastern Regions. The presence of the mutant and protective allele at a frequency of 0.5% in this region may explain the low HIV prevalence observed. In host cell infection mechanisms, HIV recruits a CCR5 coreceptor in addition to the CD4 receptor to fuse with the host membrane (Kassaye et al., 2009; Weichseldorfer et al., 2022). This specific mutation of the *CCR5* co-receptor gene, designated *CCR5Δ32*, is characterized by a deletion of 32 base pairs in its coding region. This mutation results in the production of a truncated receptor that is incapable of being recruited by HIV, and hence of infection. The *CCR5 Δ32* mutation confers strong host resistance to HIV infection (Angela Covino et al., 2016). Indeed, the Berlin patient and London patient have achieved long-term remission of HIV following bone marrow transplants from donors with the *CCR5Δ32* mutation (homozygotes) (Gupta et al., 2019; Ding et al., 2021; Prator et al., 2020; Jilg and Li, 2019; Gupta et al., 2020b). Given its role in the pathogenesis of HIV infection, this mutation represents a target for antiretroviral drugs. Additionally, there is hope of a vaccine (Ensoli et al., 2021) or gene therapy (Li et al., 2022; Kaminski et al., 2016). Although this mutation is rare or absent in Africa, it is generally present in populations living in ethnically homogeneous communities, such as Caucasian populations or the Vlachet Gypsies of Hungary (Juhász et al., 2012; Ongadi et al., 2018). This observation may provide an explanation for the presence of this genotype in the Moudang and Massas, who live in ethnically homogeneous communities.

*CCR2-64I* frequency was 12.76%, 67.71% and 19.53% for wild-type, heterozygous and mutant genotypes respectively with an allelic frequency of the protective mutation of 36.46% (Table 5). The allelic frequency of the *CCR2-64I* genotype is statistically similar to those studied in 7 ethnic groups in Cameroon, where the proportion ranged from 10.8% to 31.3% (Ma et al., 2005). In a population of children and adolescents living with HIV in the Central Region, the heterozygous *CCR2-64I* genotype was found in 10.25%, 48.93%, and 80% of rapid progressors (RP), slow progressors (SP), and long-term non-progressors (LTNP), respectively, with a significant difference between rapid progressors and slow progressors (*p = 0.0001*) and slow progressors and long-term non-progressors LTNP (*p = 0.0002*), suggesting a protective effect of the mutant allele (DHS cameroon, 2018). The same study showed that the homozygous *CCR2-64I* protective gene was present only in slow progressors at 2.12% compared with 19.53% in our study (*p = 0.003*). Moreover, the homozygous *CCR2-64I* protective gene was present at 17.6% in the study conducted by Nkenfou et al., in 2013 in the West Cameroon Region (Sawyer et al., 2006). Studies around the world have shown the protective effects of this mutation in the acquisition or progression to the AIDS stage certain populations (Köksal et al., 2021; Apostolakis et al., 2005; Farissi et al., 2020). Nevertheless, a study conducted in Korea demonstrated that this mutation is associated with a rapid progression to AIDS or death, contingent on the stage of HIV infection and interactions with other genetic variants (Choi et al., 2007).

The frequencies observed for the *SDF1* genotypes were 10.94%, 85.16%, and 3.9%, respectively, for wildtype, heterozygous, and mutant genotypes. The allelic frequency of the *SDF1-3′A* protective mutation was 25.19%, which was significantly higher than in studies carried out in the Western and Central Regions or in seven ethnic groups in Cameroon (*p < 0.001*) (Nakajima et al., 2009; Javanbakht et al., 2006; Goldschmidt et al., 2006). *SDF1* codes for a ligand of the CXCR4 receptor, and its presence is linked to either delayed progression of AIDS or rapid progression of the disease and death. Additionally, it has been shown to influence the onset of known neurodegenerative diseases (Guerini et al., 2008). SDF1 is a ligand of the CXCR4 receptor that has been demonstrated to influence the progression to the AIDS stage. The *SDF-1* gene is distinguished by the substitution of guanine for adenine at position 801 at the 3′untranslated end of the *SDF-1* gene (Agrawal et al., 2004). The global, regional, and ethnic frequency distributions of SDF1 genotypes and the *SDF1-3′-A* allele exhibit considerable variability (Reiche et al., 2006; Modi et al., 2005; Ding et al., 2018). Although this mutation rate is significant, it is within the range found in most studies conducted in Africa and around the world over the years (Su et al., 1999; Tan et al., 2010).

The frequencies of *59029 A/G CCR5* promoter genotypes were 12.24%; 66.93%; 20.83% for wildtype, heterozygous and mutant. The mutated promoter gene, with an allele frequency of 37.56%, was significantly higher than that observed in other studies in Cameroon (*p* < 0.08). The *CCR5-G59029* allele is characterized by a substitution of A-G in the promoter region, resulting in decreased promoter activity and reduced coreceptor expression (Mehlotra et al., 2015). Individuals homozygous for the *CCR5-G59029* gene progress to AIDS on average 3.8 years slower than individuals heterozygous for *CCR5A59029* (Gornalusse et al., 2015). In fact, some recent data show regulation of CD4^+^ T lymphocyte apoptosis induced by expression of genes for this co-receptor in patients infected with HIV (Joshi et al., 2017). More specifically, the *CCR5-A59029* mutation has been shown to slow down chemokine co-receptor gene expression by inhibiting the recruitment of transcription factors (Jiang et al., 2011) or insensitive to transcriptional activation by HIV (Gornalusse et al., 2015). The presence of this mutation could perhaps explain the low prevalence of HIV in these two regions of Cameroon, in line with other studies conducted worldwide (Mehlotra et al., 2015; Rathore et al., 2009; Coloccini et al., 2014).

The frequency of genotypes of *Trim5α* gene was 3.12%, 36.72%, and 60.16% for wild-type, heterozygous, and mutant genotypes, respectively, with an allelic frequency of 69.33%. This mutation was significantly higher in the northern population compared to the central population where the allelic frequency was 42.26% (*p < 0.001*) (Javanbakht et al., 2006). The tripartite interaction motif 5α (Trim5α) is recognized as an inhibitory factor blocking infections by retroviruses such as HIV. It is a factor that can interact with RNA just after decapsidation, forming an addressed proteasome capable of being recognized and digested (Kim et al., 2019; Anderson et al., 2006). This genotype has been identified in several studies as an innate factor restricting HIV infection (Gupta and Padh, 2012; Jang et al., 2008). This mutation could also be associated with the low prevalence of HIV in these two regions of Cameroon.

Overall, protective ARGs interact directly with HIV during the early stages of infection. Their natural antiviral activities are more effective when these genes are present in the majority of individuals in a given population. While homozygous *CCR5∆32* individuals are protected against viral infection, heterozygotes progress very slowly towards the AIDS stage. So the more an individual has a variety of protective genes, the more the infection is inhibited and the progression of the disease to AIDS is slowed.

The slow progression of infection to the final stage has implications for the rate of spread and prevalence of HIV. In fact, the rapid progression of HIV to the final stage has been associated with an increase in virulence and pathogenicity (Coloccini et al., 2014; Anderson et al., 2006). In HIV + patients in the AIDS stage, viral tropism has evolved from R5 to R4 or both, meaning that the virus can infect a wide range of immune cells with an increased viral load (Naif, 2013), which favors transmission. HIV transmission depends on donor viral load, viral tropism, and host immunity, among other factors (Anderson et al., 2006; Fraser et al., 2014). Thus, a more virulent transmitted virus will evolve more rapidly in a population that does not have strong immunogenic resistance.

It is important to consider the potential implications of conducting a gene therapy clinical trial in a population that does not have a high frequency of protective genes (ARGs) (Casado et al., 2020). This could result in immunological pressure on the virus, which may lead to viral escape through a change in tropism, from R5 to R4 or R5/R4, or even CCR2 (Islam et al., 2021). Indeed, the potential for viral escape has implications beyond mere therapeutic failure. More significantly, it could result in the selection of pathogenic and virulent mutations (Arif et al., 2017; Johnson et al., 2022). It is therefore suggested to conduct a clinical trial on a population with a high allelic frequency of protective genes. The current HIV treatments are designed to limit viral replication; however, stopping them immediately results in a rebound in viral replication (Bailón et al., 2022; De Scheerder et al., 2019). Gene therapy, as evidenced by the London and Berlin patients, mimicking this using the CRISPR-Cas9 system in a population that is already resistant to HIV infection, could prove to be a viable solution.

The data provided by ARGs are of interest for gene therapy planning because it would be more interesting and effective to conduct clinical trials in a population with a high frequency of protective genes. Modern gene therapies use gene-editing molecular scissors such as the CRISPR-Cas9 system (Bhowmik and Chaubey, 2022; Akram et al., 2023) or ZFN (Didigu et al., 2014; Ishida, 2020). These molecular scissors can be used to induce *CCR5∆32* or *CCR5proA/G* mutations in a population at risk but with a high frequency of other protective genes. However, given the security problems that have prevailed in these regions of northern Cameroon for several years, the influx of refugees (BANQUE MONDIALE) could affect the gene pool by altering the Harding-Weinberg equilibrium of protective ARGs. Three hypotheses may be posited regarding the potential impact of the influx of refugees on the genetic structure of ARGs in these regions. The initial hypothesis is that the refugees will reduce the allelic frequency of the ARGs prior to the commencement of a clinical trial. The consequence would be a high probability of viral escape, therapeutic failure, and, at worst, a selection mutation. The second desirable scenario would be to carry out the same study in refugee populations, to measure the allele frequency of ARG genes before a clinical trial. The third hypothesis is probable that the genetic impact of refugees will be insignificant, given that they exhibited comparable socio-cultural traits to those of the northern Cameroon.

The therapeutic potential of gene therapy will be the subject of several studies over the coming years. The objective is to describe the genetic structure of the entire target population and the associated immuno-genetic mechanisms in several scenarios.

### Limits of the study

The size of our study population was not large enough. A larger study would yield more conclusive results by incorporating ethnicity as a variable.

*HLA-B* gene variants, particularly *HLA-B27* and *HLA-B57* (Loffredo et al., 2009), have been demonstrated to exhibit resistance to HIV in individuals who possess these variants. Further investigation into the role of additional genetic factors, such as *HLA* genes, as well as defensins gene (Lafferty et al., 2017), *KIR* (Carr et al., 2007), *APOBEC3′A* (McDonnell et al., 2021), and others (He and Wu, 2019) (miRNA through epigenetics), in HIV resistance would be of significant interest.

## Conclusion

The aim of this study was to understand the low prevalence of HIV in the North and Far North regions of Cameroon. It seems that the five genes associated with HIV infection (*CCR5Δ32, CCR2-64I, SDF1-3ʹA, CCR5 promoter 59029 A/G* and *TRIM 5α138Q*) could explain the low prevalence of HIV infection in these two regions. These genes were found at a higher frequency in these two regions compared to the other regions of Cameroon and can explain the low prevalence of HIV in the North of Cameroon. Knowing that HIV infection is the result of a combination of factors, monitoring of the epidemic should be continuous and even intensified, given the frequent displacement of refugees due to political insecurity, which can weaken the health system and jeopardize efforts to eliminate HIV.

In the perspectives of our work where we will be looking for mutation *CCR5∆32* in Moudang and Massa ethnic groups.

## Data Availability

The original contributions presented in the study are included in the article/supplementary material, further inquiries can be directed to the corresponding author.

## Abbreviations

ARV, Antiretroviral HIV drugs; ARG, AIDS Related Genes; AIDS, Acquired immunodeficiency syndrome; CCR5 or CD195, C-C chemokine receptor type 5; CRISPR-, Cluster of Regularly Interspaced Short Palindromic Repeats-; Cas9, *CRISPR*-associated 9; HIV, Human immunodeficiency virus; SDF1, Stromal Derivated Factor-1; Trim 5α, Tripartite motif-containing protein 5alpha; ZFN, Zinc Finger.

## References

[B1] Abrahams M. R. Anderson J. A. Giorgi E. E. Seoighe C. Mlisana K. Ping L. H. (2009). Quantitating the multiplicity of infection with human immunodeficiency virus type 1 subtype C reveals a non-Poisson distribution of transmitted variants. J. Virol. 83 (8), 3556–3567. 10.1128/JVI.02132-08 19193811 PMC2663249

[B2] Agrawal L. Lu X. Qingwen J. VanHorn-Ali Z. Nicolescu I. V. McDermott D. H. (2004). Role for CCR5Delta32 protein in resistance to R5, R5X4, and X4 human immunodeficiency virus type 1 in primary CD4+ cells. J. Virol. 78 (5), 2277–2287. 10.1128/jvi.78.5.2277-2287.2004 14963124 PMC369216

[B3] Ahmed R. A. Adekoya K. O. Onwuamah C. K. Oboh B. O. Iyer S. S. Oluwatosin A. S. (2022). Mechanism of viral suppression among HIV elite controllers and long-term nonprogressors in Nigeria and South Africa. Viruses 14 (6), 1270. 10.3390/v14061270 35746741 PMC9228396

[B4] Akram F. Sahreen S. Aamir F. Haq I. ul Malik K. Imtiaz M. (2023). An insight into modern targeted genome-editing technologies with a special focus on CRISPR/Cas9 and its applications. Mol. Biotechnol. 65, 227–242. 10.1007/s12033-022-00501-4 35474409 PMC9041284

[B5] Ammaranond P. van Bockel D. J. Petoumenos K. McMurchie M. Finlayson R. Middleton M. G. (2011). HIV immune escape at an immunodominant epitope in HLA-B*27–Positive individuals predicts viral load outcome. J. Immunol. 186, 479–488. 10.4049/jimmunol.0903227 21115730

[B6] Anderson J. L. Campbell E. M. Wu X. Vandegraaff N. Engelman A. Hope T. J. (2006). Proteasome inhibition reveals that a functional preintegration complex intermediate can Be generated during restriction by diverse TRIM5 proteins. J. Virol. 80 (19), 9754–9760. 10.1128/JVI.01052-06 16973579 PMC1617233

[B7] Angela Covino D. Sabbatucci M. Fantuzzi L. (2016). The CCL2/CCR2 Axis in the pathogenesis of HIV-1 infection: a new cellular target for therapy? Curr. Drug Targets 17 (1), 76–110. 10.2174/138945011701151217110917 26687605

[B8] Apostolakis S. Baritaki S. Krambovitis E. Spandidos D. A. (2005). Distribution of HIV/AIDS protective SDF1, CCR5 and CCR2 gene variants within Cretan population. J. Clin. Virol. 34 (4), 310–314. 10.1016/j.jcv.2005.01.010 16286055

[B9] Arenzana-Seisdedos F. (2015). SDF-1/CXCL12: a chemokine in the life cycle of HIV. Front. Immunol. 6, 256. 10.3389/fimmu.2015.00256 26097474 PMC4456947

[B10] Arif M. S. Hunter J. Léda A. R. Zukurov J. P. L. Samer S. Camargo M. (2017). Pace of coreceptor tropism switch in HIV-1-Infected individuals after recent infection. J. Virol. . 12 Sept. 91 (19). 10.1128/jvi.00793-17 PMC5599763 28659473

[B11] Bailón L. Llano A. Cedeño S. Escribà T. Rosás-Umbert M. Parera M. (2022). Safety, immunogenicity and effect on viral rebound of HTI vaccines in early treated HIV-1 infection: a randomized, placebo-controlled phase 1 trial. Nat. Med. 28, 2611–2621. 10.1038/s41591-022-02060-2 36302893

[B12] BANQUE MONDIALE. World Bank. 2023. Cameroun - vue d’ensemble. Available at: https://www.banquemondiale.org/fr/country/cameroon/overview.

[B13] Bhowmik R. Chaubey B. (2022). CRISPR/Cas9: a tool to eradicate HIV-1. AIDS Res. Ther. 1 déc 19 (1), 58. 10.1186/s12981-022-00483-y PMC9713993 36457057

[B14] Brambilla A. Villa C. Rizzardi G. Veglia F. Ghezzi S. Lazzarin A. (2000). Shorter survival of SDF1-3′A/3′A homozygotes linked to CD4+ T cell decrease in advanced human immunodeficiency virus type 1 infection. J. Infect. Dis. 182 (1), 311–315. 10.1086/315650 10882614

[B15] Burgoyne R. W. Tan D. H. S. (2008). Prolongation and quality of life for HIV-infected adults treated with highly active antiretroviral therapy (HAART): a balancing act. J. Antimicrob. Chemother. 61 (3), 469–473. 10.1093/jac/dkm499 18174196

[B16] Burton C. T. Gotch F. M. Imami N. (2005). CCR2/64I mutation detection in a HIV-1-positive patient with slow CD4 T-cell decline and delay in disease progression. Int. J. STD AIDS 16 (5), 392–394. 10.1258/0956462053888817 15949075

[B17] Carr W. H. Rosen D. B. Arase H. Nixon D. F. Michaelsson J. Lanier L. L. (2007). Cutting edge: *KIR3DS1*, a gene implicated in resistance to progression to AIDS, encodes a DAP12-associated receptor expressed on NK cells that triggers NK cell activation. J. Immunol. 178, 647–651. 10.4049/jimmunol.178.2.647 17202323 PMC2561215

[B18] Casado C. Galvez C. Pernas M. Tarancon-Diez L. Rodriguez C. Sanchez-Merino V. (2020). Permanent control of HIV-1 pathogenesis in exceptional elite controllers: a model of spontaneous cure. Sci. Rep. 10 (1), 1902. 10.1038/s41598-020-58696-y 32024974 PMC7002478

[B19] Chan E. Towers G. J. Qasim W. (2014). Gene therapy strategies to exploit TRIM derived restriction factors against HIV-1. Viruses 6 (1), 243–263. 10.3390/v6010243 24424502 PMC3917441

[B20] Choi B. S. Choi J. H. Kim S. S. Kee M. K. Lee J. S. (2007). CCR2b-64I allelic polymorphisms in advanced HIV-infected Koreans accelerate disease progression. AIDS Res. Hum. Retroviruses 23 (6), 805–811. 10.1089/aid.2006.0133 17604544

[B21] Coloccini R. S. Dilernia D. Ghiglione Y. Turk G. Laufer N. Rubio A. (2014). Host genetic factors associated with symptomatic primary HIV infection and disease progression among argentinean seroconverters. PLOS ONE 9, e113146. 10.1371/journal.pone.0113146 25406087 PMC4236131

[B22] Dambaya B. Nkenfou C. N. Mekue L. Této G. Ngoufack N. Ambada G. (2019). TRIM5α 136Q, CCR5 promoter 59029G and CCR264I alleles impact the progression of HIV in children and adolescents. Appl. Clin. Genet. 12, 203–211. 10.2147/TACG.S205335 31807050 PMC6844200

[B23] De Scheerder M. A. Vrancken B. Dellicour S. Schlub T. Lee E. Shao W. (2019). HIV rebound is predominantly fueled by genetically identical viral expansions from diverse reservoirs. Cell Host Microbe 26, 347–358. 10.1016/j.chom.2019.08.003 31471273 PMC11021134

[B24] DHS cameroon (2018). Demographic and health survey Cameroon. Available at: https://dhsprogram.com/pubs/pdf/OF44/OF44.HIV.E.pdf.

[B25] Didigu C. A. Wilen C. B. Wang J. Duong J. Secreto A. J. Danet-Desnoyers G. A. (2014). Simultaneous zinc-finger nuclease editing of the HIV coreceptors ccr5 and cxcr4 protects CD4+ T cells from HIV-1 infection. Blood 123 (1), 61–69. 10.1182/blood-2013-08-521229 24162716 PMC3879906

[B26] Ding J. Liu Y. Lai Y. (2021). Knowledge from London and Berlin: finding threads to a functional HIV cure. Front. Immunol. 12, 688747. 10.3389/fimmu.2021.688747 34122453 PMC8190402

[B27] Ding J. Zhao J. Zhou J. Li X. Wu Y. Ge M. (2018). Association of gene polymorphism of SDF1(CXCR12) with susceptibility to HIV-1 infection and AIDS disease progression: a meta-analysis. PLOS ONE 13, e0191930. 10.1371/journal.pone.0191930 29420545 PMC5805253

[B28] Ensoli B. Moretti S. Borsetti A. Maggiorella M. T. Buttò S. Picconi O. (2021). New insights into pathogenesis point to HIV-1 Tat as a key vaccine target. Arch. Virol. 166, 2955–2974. 10.1007/s00705-021-05158-z 34390393 PMC8363864

[B29] Farissi F. Z. El Annaz H. Melloul M. El Alaoui M. A. Tagajdid M. R. Abi R. (2020). Analysis of the CCR2-64I (rs1799864) genetic polymorphism distribution and its effect on the risk of HIV-1 infection and immunovirological outcomes in Moroccan ART-treated individuals. Gene Rep. 20, 100715. 10.1016/j.genrep.2020.100715

[B30] Fraser C. Lythgoe K. Leventhal G. E. Shirreff G. Hollingsworth T. D. Alizon S. (2014). Virulence and pathogenesis of HIV-1 infection: an evolutionary perspective. Science 343, 1243727. 10.1126/science.1243727 24653038 PMC5034889

[B31] Goldschmidt V. Bleiber G. May M. Martinez R. Ortiz M. Telenti A. (2006). Role of common human TRIM5alpha variants in HIV-1 disease progression. Retrovirology 3, 54. 10.1186/1742-4690-3-54 16925802 PMC1560158

[B32] Gornalusse G. G. Mummidi S. Gaitan A. A. Jimenez F. Ramsuran V. Picton A. (2015). Epigenetic mechanisms, T-cell activation, and CCR5 genetics interact to regulate T-cell expression of CCR5, the major HIV-1 coreceptor. Proc. Natl. Acad. Sci. 10.1073/pnas.1423228112 PMC4553789 26307764

[B33] Guerini F. R. Delbue S. Zanzottera M. Agliardi C. Saresella M. Mancuso R. (2008). Analysis of CCR5, CCR2, SDF1 and RANTES gene polymorphisms in subjects with HIV-related PML and not determined leukoencephalopathy. Biomed. Pharmacother. 62, 26–30. 10.1016/j.biopha.2007.04.005 17560067

[B34] Gupta A. Padh H. (2012). The global distribution of CCR5 delta 32 polymorphism: role in HIV-1 protection. BMC Infect. Dis. 12, O16. 10.1186/1471-2334-12-S1-O16

[B35] Gupta R. K. Abdul-Jawad S. McCoy L. E. Mok H. P. Peppa D. Salgado M. (2019). HIV-1 remission following CCR5Δ32/Δ32 haematopoietic stem-cell transplantation. Nature 568 (7751), 244–248. 10.1038/s41586-019-1027-4 30836379 PMC7275870

[B36] Gupta R. K. Peppa D. Hill A. L. Gálvez C. Salgado M. Pace M. (2020a). Evidence for HIV-1 cure after CCR5Δ32/Δ32 allogeneic haemopoietic stem-cell transplantation 30 months post analytical treatment interruption: a case report. Lancet HIV 7 (5), e340–e347. 10.1016/S2352-3018(20)30069-2 32169158 PMC7606918

[B37] Gupta R. K. Peppa D. Hill A. L. Gálvez C. Salgado M. Pace M. (2020b). Evidence for HIV-1 cure after CCR5Δ32/Δ32 allogeneic haemopoietic stem-cell transplantation 30 months post analytical treatment interruption: a case report. Lancet HIV 7 (5), e340–e347. 10.1016/S2352-3018(20)30069-2 32169158 PMC7606918

[B38] He S. Wu Y. (2019). Relationships between HIV-mediated chemokine coreceptor signaling, cofilin hyperactivation, viral tropism switch and HIV-mediated CD4 depletion. Curr. HIV Res. 1 déc 17 (6), 388–396. 10.2174/1570162X17666191106112018 31702526

[B39] Ishida T. (2020). Virucidal activities of zinc-finger antiviral proteins and zinc-binding domains for virus entry, DNA/RNA replication and spread. Edelweiss J. Biomed. Res. Rev. 10.33805/2690-2613.109

[B40] Islam S. Moni M. A. Urmi U. L. Tanaka A. Hoshino H. (2021). C-C Chemokine receptor-like 2 (CCRL2) acts as coreceptor for human immunodeficiency virus-2. Brief. Bioinform 22, bbaa333. 10.1093/bib/bbaa333 33253374

[B41] Jang D. H. Choi B. S. Kim S. S. (2008). The effects of RANTES/CCR5 promoter polymorphisms on HIV disease progression in HIV-infected Koreans. Int. J. Immunogenet 35, 101–105. 10.1111/j.1744-313X.2007.00743.x 18218038

[B42] Javanbakht H. An P. Gold B. Petersen D. C. O’Huigin C. Nelson G. W. (2006). Effects of human TRIM5alpha polymorphisms on antiretroviral function and susceptibility to human immunodeficiency virus infection. Virology 354, 15–27. 10.1016/j.virol.2006.06.031 16887163

[B43] Jiang D. Mummidi S. Ahuja S. K. Jarrett H. W. (2011). CCR5 promoter haplotype transcription complex characterization. J. Health Care Poor Underserved 22, 73–90. 10.1353/hpu.2011.0169 22102307 PMC3326658

[B44] Jilg N. Li J. Z. (2019). On the Road to a HIV cure: moving beyond Berlin and London. Infect. Dis. Clin. 33 (3), 857–868. 10.1016/j.idc.2019.04.007 PMC6814144 31395147

[B45] Johnson M. M. Jones C. E. Clark D. N. (2022). The effect of treatment-associated mutations on HIV replication and transmission cycles. Viruses 15, 107. 10.3390/v15010107 36680147 PMC9861436

[B46] Joshi A. Punke E. B. Sedano M. Beauchamp B. Patel R. Hossenlopp C. (2017). CCR5 promoter activity correlates with HIV disease progression by regulating CCR5 cell surface expression and CD4 T cell apoptosis. Sci. Rep. 7, 232. 10.1038/s41598-017-00192-x 28331180 PMC5427887

[B47] Juhász E. Béres J. Kanizsai S. Nagy K. (2012). The consequence of a founder effect: CCR5-∆32, CCR2-64I and SDF1-3’A polymorphism in vlach gypsy population in Hungary. Pathol. Oncol. Res. 18 (2), 177–182. 10.1007/s12253-011-9425-4 21667221

[B48] Kaminski R. Bella R. Yin C. Otte J. Ferrante P. Gendelman H. E. (2016). Excision of HIV-1 DNA by gene editing: a proof-of-concept *in vivo* study. Gene Ther. 23 (8), 690–695. 10.1038/gt.2016.41 27194423 PMC4974122

[B49] Kassaye S. Johnston E. McColgan B. Kantor R. Zijenah L. Katzenstein D.(2009). Envelope coreceptor tropism, drug resistance, and viral evolution among subtype C HIV-1-Infected individuals receiving nonsuppressive antiretroviral therapy. JAIDS J. Acquir Immune Defic. Syndr. 10.1097/QAI.0b013e31818ffdff PMC2818215 19295330

[B50] Kim K. Dauphin A. Komurlu S. McCauley S. M. Yurkovetskiy L. Carbone C. (2019). Cyclophilin A protects HIV-1 from restriction by human TRIM5α. Nat. Microbiol. 4 (12), 2044–2051. 10.1038/s41564-019-0592-5 31636416 PMC6879858

[B51] Köksal M. O. Akgül B. Beka H. Çiftçi S. Keskin F. Eraksoy H. (2021). Frequency of CCR5-Δ32, CCR2-64I and SDF1-3’A alleles in HIV-infected and uninfected patients in Istanbul, Turkey. J. Infect. Dev. Ctries. 15, 1183–1189. 10.3855/jidc.12861 34516427

[B52] Kristiansen T. B. Knudsen T. B. Ohlendorff S. Eugen-Olsen J. (2001). A new multiplex PCR strategy for the simultaneous determination of four genetic polymorphisms affecting HIV-1 disease progression. J. Immunol. Methods 252, 147–151. 10.1016/s0022-1759(01)00349-0 11334974

[B53] Lafferty M. Sun L. Christensen-Quick A. Lu W. Garzino-Demo A. (2017). Human beta defensin 2 selectively inhibits HIV-1 in highly permissive CCR6⁺CD4⁺ T cells. Viruses 9 (5), 111. 10.3390/v9050111 28509877 PMC5454423

[B54] Li S. Holguin L. Burnett J. C. (2022). CRISPR-Cas9-mediated gene disruption of HIV-1 co-receptors confers broad resistance to infection in human T cells and humanized mice. Mol. Ther. - Methods Clin. Dev. 24, 321–331. 10.1016/j.omtm.2022.01.012 35229006 PMC8847835

[B55] Loffredo J. T. Sidney J. Bean A. T. Beal D. R. Bardet W. Wahl A. (2009). Two MHC class I molecules associated with elite control of immunodeficiency virus replication, mamu-B*08 and HLA-B*2705, bind peptides with sequence similarity. J. Immunol. 182, 7763–7775. 10.4049/jimmunol.0900111 19494300 PMC2701622

[B56] Lot F. (2008). Épidémiologie du VIH/sida et des autres infections sexuellement transmissibles chez les femmes. médecine/sciences 24, 7–19. 10.1051/medsci/2008242s7 18681115

[B57] Lwanga S. K. Lemeshow S. Organization W. H. (1991). Détermination de la taille d’ un échantillon dans les études sanométriques: manuel pratique. Organ. Mond. Santé. Available at: https://apps.who.int/iris/handle/10665/36881.

[B58] Ma L. Marmor M. Zhong P. Ewane L. Su B. Nyambi P. (2005). Distribution of CCR2-64I and SDF1-3′A alleles and HIV status in 7 ethnic populations of Cameroon. JAIDS J. Acquir Immune Defic. Syndr. 10.1097/01.qai.0000157008.66584.d6 16123688

[B59] Manen D. van Rits M. A. N. Beugeling C. Dort K. van Schuitemaker H. Kootstra N. A. (2008). The effect of Trim5 polymorphisms on the clinical course of HIV-1 infection. PLOS Pathog. 10.1371/journal.ppat.0040018 PMC2222955 18248091

[B60] Mbopi-Keou F. X. Nguefack-Tsague G. Mireille G. C. Abo’o Abessolo S. Angwafo I. I. I. F. Muna W. (2014). Facteurs de risque de l’infection par le VIH dans le district de santé de Meyomessala au Cameroun. Pan Afr. Med. J. 18. 10.11604/pamj.2014.18.161.3238 PMC4236842 25419299

[B61] McDonnell M. M. Karvonen S. C. Gaba A. Flath B. Chelico L. Emerman M. (2021). Highly-potent, synthetic APOBEC3s restrict HIV-1 through deamination-independent mechanisms. PLOS Pathog. 17, e1009523. 10.1371/journal.ppat.1009523 34170969 PMC8266076

[B62] Mehlotra R. K. Hall N. B. Bruse S. E. John B. Blood Zikursh M. J. Stein C. M. (2015). *CCR2, CCR5*, and *CXCL12* variation and HIV/AIDS in Papua New Guinea. Infect. Genet. Evol. 36, 165–173. 10.1016/j.meegid.2015.09.014 26397046 PMC4644711

[B63] Mellado M. Rodríguez-Frade J. M. Vila-Coro A. J. de Ana A. M. Martínez A. C. (1999). Chemokine control of HIV-1 infection. Nature 400, 723–724. 10.1038/23382 10466720

[B64] Metodiev K. (2012). Immunodeficiency. London, Unite Kingdom: IntechOpen Limited. BoD – Books on Demand, 406.

[B65] Mishra D. O`Laughlin K. Spiegel P. (2021). A systematic review evaluating HIV prevalence among conflict-affected populations, 2005-2020. Aids Rev. 23 (3), 6530. 10.24875/aidsrev.200001311 PMC9478562 34279517

[B66] Modi W. S. Scott K. Goedert J. J. Vlahov D. Buchbinder S. Detels R. (2005). Haplotype analysis of the SDF-1 (CXCL12) gene in a longitudinal HIV-1/AIDS cohort study. Genes Immun. 6, 691–698. 10.1038/sj.gene.6364258 16177829

[B67] Naif H. M. (2013). Pathogenesis of HIV infection. Infect. Dis. Rep. 5, e6. 10.4081/idr.2013.s1.e6 PMC3892619 24470970

[B68] Nakajima T. Nakayama E. E. Kaur G. Terunuma H. Mimaya J. ich Ohtani H. (2009). Impact of novel TRIM5alpha variants, Gly110Arg and G176del, on the anti-HIV-1 activity and the susceptibility to HIV-1 infection. AIDS 23, 2091–2100. 10.1097/QAD.0b013e328331567a 19710594

[B69] Nkenfou C. Njolle N. E. Nguefeu C. Moudourou S. A. Fainguem N. Fokam J. (2018). Syphilis is on the rise among pregnant women in Cameroon. J. Virus Erad. 4, 36–37. 10.1016/s2055-6640(20)30432-5

[B70] Nkenfou C. N. Mekue L. C. M. Nana C. T. Kuiate J. R. (2013). Distribution of CCR5-Delta32, CCR5 promoter 59029 A/G, CCR2-64I and SDF1-3’A genetic polymorphisms in HIV-1 infected and uninfected patients in the West Region of Cameroon. BMC Res. Notes 6 (1), 288. 10.1186/1756-0500-6-288 23880174 PMC3733889

[B71] Nkenfou C. N. Tchakounté C. Nkenfou-Tchinda C. N. Ngoufack M. N. Yatchou L. G. Elong E. (2021). Protective effect of the combination of wild-type genotypes (G/G and G/G) of CCR2-64V and SDF1-3A’ genes in serodiscordant couples in yaounde-Cameroon. Curr. HIV Res. 1 juill 19 (4), 342–351. 10.2174/1570162X19666210412121143 33845725

[B72] Nyambi P. Zekeng L. Kenfack H. Tongo M. Nanfack A. Nkombe I. (2002). HIV infection in rural villages of Cameroon. JAIDS J. Acquir Immune Defic. Syndr. 10.1097/00126334-200212150-00008 12473839

[B73] Ongadi B. A. Obiero G. Lihana R. W. Kiiru J. N. (2018). Distribution of genetic polymorphism in the CCR5 among caucasians, asians and africans: a systematic review and meta-analysis. Open J. Genet. 08(03):54. 10.4236/ojgen.2018.83006

[B74] Prator C. A. Donatelli J. Henrich T. J. (2020). From Berlin to London: HIV-1 reservoir reduction following stem cell transplantation. Curr. HIV/AIDS Rep. 17 (4), 385–393. 10.1007/s11904-020-00505-2 32519184

[B75] Rathore A. Chatterjee A. Sivarama P. Yamamoto N. Singhal P. K. Dhole T. N. (2009). Association of CCR5-59029 A/G and CCL3L1 copy number polymorphism with HIV type 1 transmission/progression among HIV type 1-seropositive and repeatedly sexually exposed HIV type 1-seronegative North Indians. AIDS Res. Hum. Retroviruses 25 (11), 1149–1156. 10.1089/aid.2008.0019 19886839

[B76] Reiche E. M. V. Watanabe M. a. E. Bonametti A. M. Morimoto H. K. Morimoto A. A. Wiechmann S. L. (2006). Stromal cell-derived factor 1 (SDF1) genetic polymorphism in a sample of healthy individuals, seronegative individuals exposed to human immunodeficiency virus type 1 (HIV-1) and patients infected with HIV-1 from the Brazilian population. Int. J. Immunogenet 33 (2), 127–133. 10.1111/j.1744-313X.2006.00583.x 16611258

[B77] Sadri F. Rezaei Z. Fereidouni M. (2022). The significance of the SDF-1/CXCR4 signaling pathway in the normal development. Mol. Biol. Rep. 49 (4), 3307–3320. 10.1007/s11033-021-07069-3 35067815

[B78] Sawyer S. L. Wu L. I. Akey J. M. Emerman M. Malik H. S. (2006). High-frequency persistence of an impaired allele of the retroviral defense gene TRIM5alpha in humans. Curr. Biol. Cb. 10 janv 16 (1), 95–100. 10.1016/j.cub.2005.11.045 16401428

[B79] Sayan M. Sargin F. Inan D. Sevgi D. Y. Celikbas A. K. Yasar K. (2016). HIV-1 transmitted drug resistance mutations in newly diagnosed antiretroviral-naive patients in Turkey. AIDS Res. Hum. Retroviruses 32 (1), 26–31. 10.1089/AID.2015.0110 26414663 PMC4692107

[B80] Signoret N. Oldridge J. Pelchen-Matthews A. Klasse P. J. Tran T. Brass L. F. (1997). Phorbol esters and SDF-1 induce rapid endocytosis and down modulation of the chemokine receptor CXCR4. J. Cell Biol. 139 (3), 651–664. 10.1083/jcb.139.3.651 9348282 PMC2141706

[B81] snpStats (2024). Bioconductor. snpStats. Available at: http://bioconductor.org/packages/snpStats/.

[B82] Sokolskaja E. Luban J. (2006). Cyclophilin, TRIM5, and innate immunity to HIV-1. Curr. Opin. Microbiol. 9 (4), 404–408. 10.1016/j.mib.2006.06.011 16815734

[B83] Soriano A. Martínez C. García F. Plana M. Palou E. Lejeune M. (2002). Plasma stromal cell–derived factor (SDF)-1 levels, SDF1-3′A genotype, and expression of CXCR4 on T lymphocytes: their impact on resistance to human immunodeficiency virus type 1 infection and its progression. J. Infect. Dis. 186 (7), 922–931. 10.1086/343741 12232832

[B84] Spínola H. Couto A. R. Peixoto M. J. Anagnostou P. Destro-Bisol G. Spedini G. (2011). HLA class-I diversity in Cameroon: evidence for a north-south structure of genetic variation and relationships with african populations. Ann. Hum. Genet. 75 (6), 665–677. 10.1111/j.1469-1809.2011.00672.x 21910692

[B85] Su B. Jin L. Hu F. Xiao J. Luo J. Lu D. (1999). Distribution of two HIV-1–Resistant polymorphisms (SDF1-3′A and CCR2-64I) in east asian and world populations and its implication in AIDS epidemiology. Am. J. Hum. Genet. 65, 1047–1053. 10.1086/302568 10486323 PMC1288237

[B86] Tan X. hua yu Z. J. hong Di C. rong Hu A. Yang L. Qu S. (2010). Distribution of CCR5-Δ32, CCR5m303A, CCR2-64I and SDF1-3′A in HIV-1 infected and uninfected high-risk Uighurs in Xinjiang, China. Infect. Genet. Evol. 10.4236/ojgen.2018.83006 19958843

[B87] UNAIDS (2024). Suivi mondial de la lutte contre le sida 2024 — Indicateurs et questions pour le suivi des progrès de la Déclaration politique sur le VIH et le sida de 2021. Available at: https://www.unaids.org/fr/resources/documents/2024/global-aids-monitoring-guidelines.

[B88] Weichseldorfer M. Tagaya Y. Reitz M. DeVico A. L. Latinovic O. S. (2022). Identifying CCR5 coreceptor populations permissive for HIV-1 entry and productive infection: implications for *in vivo* studies. J. Transl. Med. 20, 39. 10.1186/s12967-022-03243-8 35073923 PMC8785515

[B89] Winkler C. Modi W. Smith M. W. Nelson G. W. Wu X. Carrington M. (1998). Genetic restriction of AIDS pathogenesis by an SDF-1 chemokine gene variant. ALIVE study, hemophilia growth and development study (HGDS), multicenter AIDS cohort study (MACS), multicenter hemophilia cohort study (MHCS), San Francisco city cohort (SFCC). Science 279 (5349), 389–393. PMID: 9430590. 10.1126/science.279.5349.389 9430590

[B90] WMA The World Medical Association-Déclaration d’Helsinki de l’AMM – Principes éthiques applicables à la recherche médicale impliquant des êtres humains. Available at: https://www.wma.net/fr/policies-post/declaration-dhelsinki-de-lamm-principes-ethiques-applicables-a-la-recherche-medicale-impliquant-des-etres-humains/.

[B91] Yandrapally S. Mohareer K. Arekuti G. Vadankula G. R. Banerjee S. (2021). HIV co-receptor-tropism: cellular and molecular events behind the enigmatic co-receptor switching. Crit. Rev. Microbiol. 47 (4), 499–516. 10.1080/1040841X.2021.1902941 33900141

